# Supplementary material for: Dynamics of primary productivity in relation to submerged vegetation of a shallow, eutrophic lagoon: A field and mesocosm study
Source: PLoS One. 2021 May 6;16(5):e0247696. doi: 10.1371/journal.pone.0247696 (PMC8101763; doi:10.1371/journal.pone.0247696)
Supplement: S1 File — (DOCX) [file pone.0247696.s001.docx]

## Light and dark bottle experiment

On the 27^th^ August twelve bottles of 250 ml volume were filled in pairs from each mesocosm. One bottle was covered with aluminium foil and is referring to as the dark bottle, while the other one is called the light bottle. Both bottles were deployed into the same mesocosm were the water was taken from. The evolution of oxygen concentration was measured, after the initial oxygen levels were taken, (optode logger, Hq40, LDO, Hach-Lange) every two hours from 10:00 to 16:00 (CET). The dark bottles were kept in the mesocosm for 24h before measuring the final oxygen concentration. Percentages of oxygen saturation measured were converted in mg l^-1^ using solubility values (Benson and Krause 1984) and scaled to the bottles volume. Hourly rates of oxygen evolution were compared with hourly rates of oxygen evolution in mesocosm to cross-validate dissolved oxygen measurements. The results are shown in the supplement (S1 Fig).
