# Supplementary figures and images for: Dynamics of primary productivity in relation to submerged vegetation of a shallow, eutrophic lagoon: A field and mesocosm study
Source: PLoS One. 2021 May 6;16(5):e0247696. doi: 10.1371/journal.pone.0247696 (PMC8101763; doi:10.1371/journal.pone.0247696)

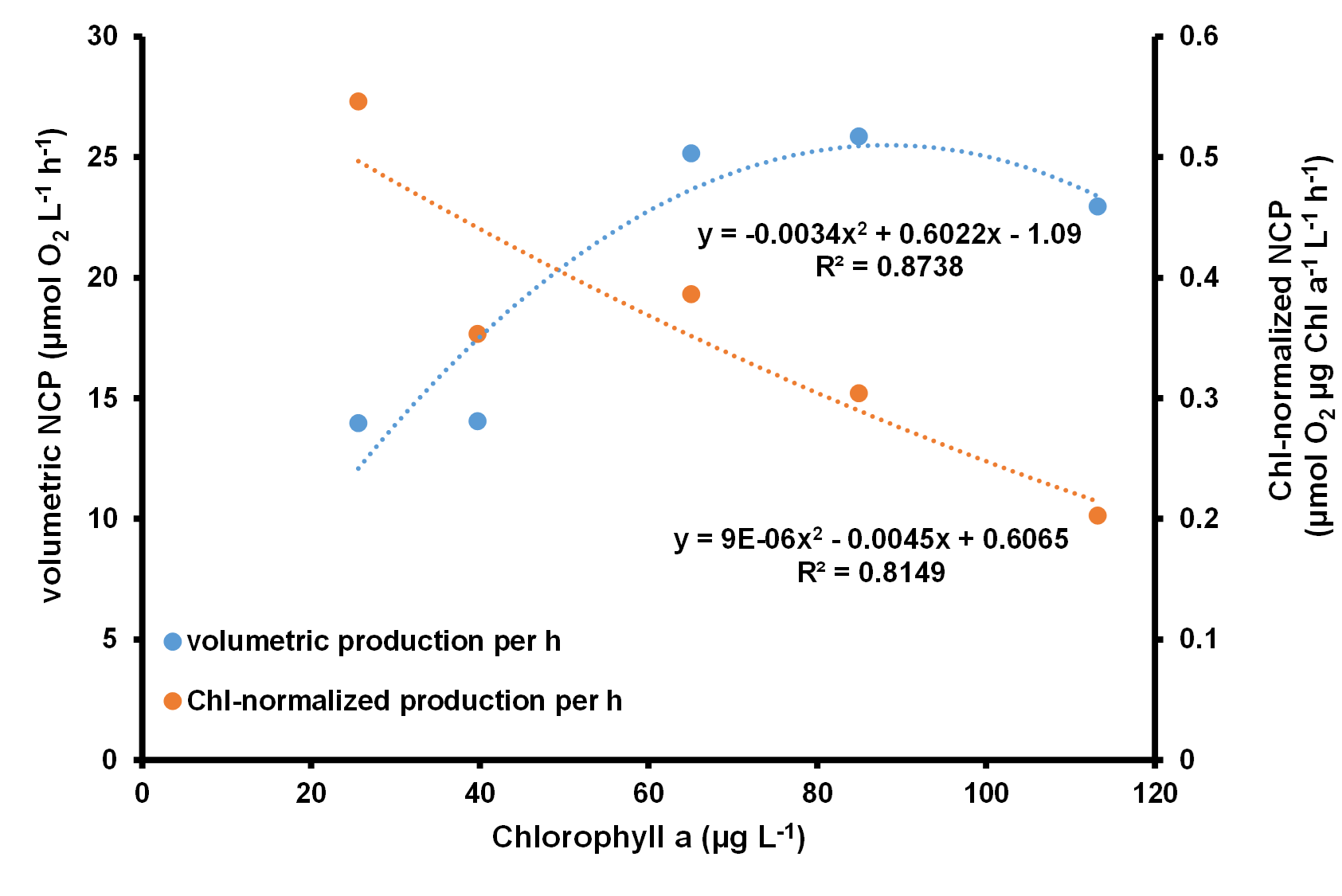

Supplement: S1 Fig — Transparent and darkened bottles were used to measure oxygen concentration after every four hours and 24 hours. One pair of bottles is missing, as the plug of a dark-bottle loosened. (TIF) [file pone.0247696.s003.tif]

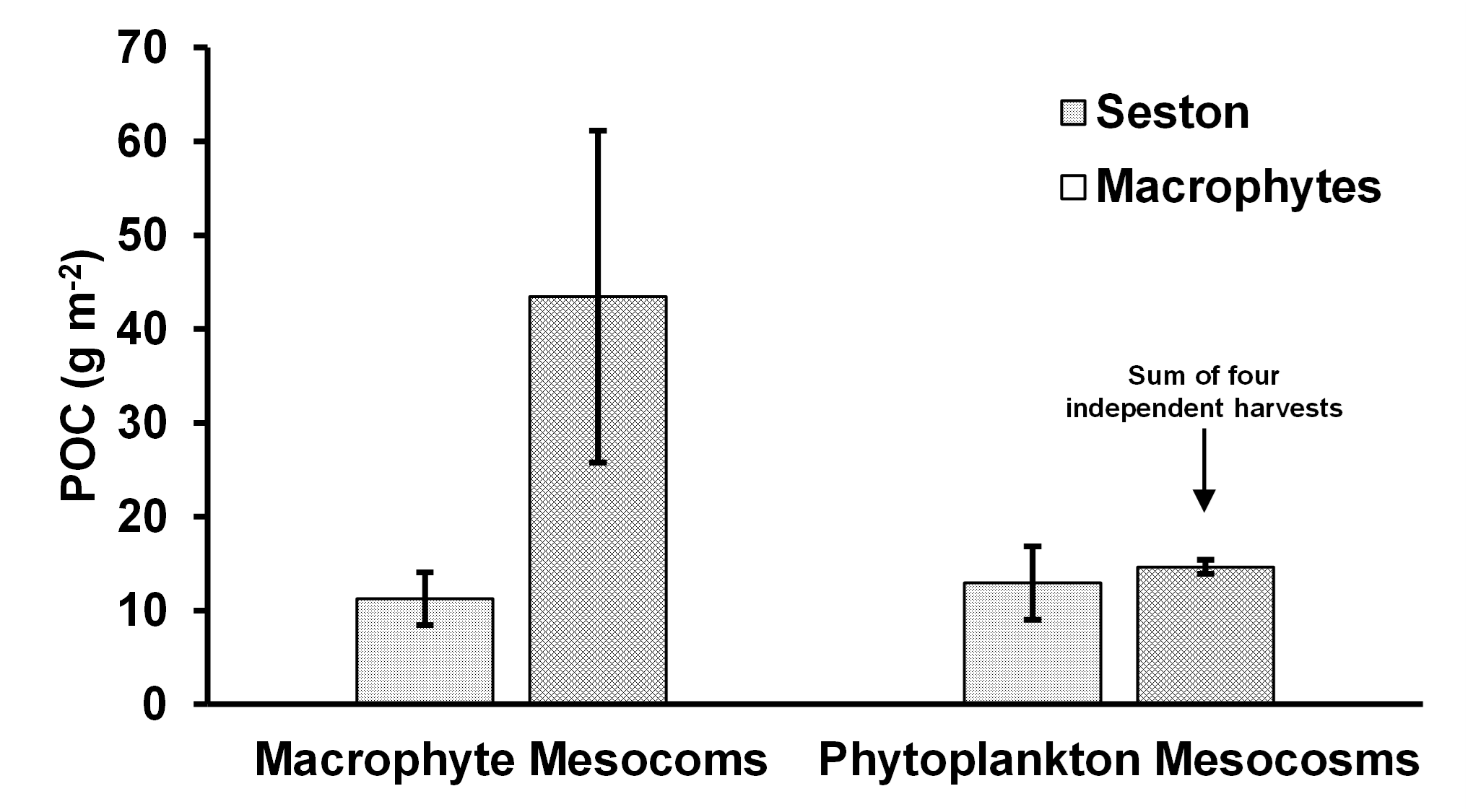

Supplement: S2 Fig — Macrophyte mesocosms were left untouched until the end of the experiment. The POC value for macrophytes in phytoplankton mesocosms represents a cumulative sum of all previous harvests. Biomass in phytoplankton mesocosms was harvested four times from experiment start to end. (TIF) [file pone.0247696.s004.tif]

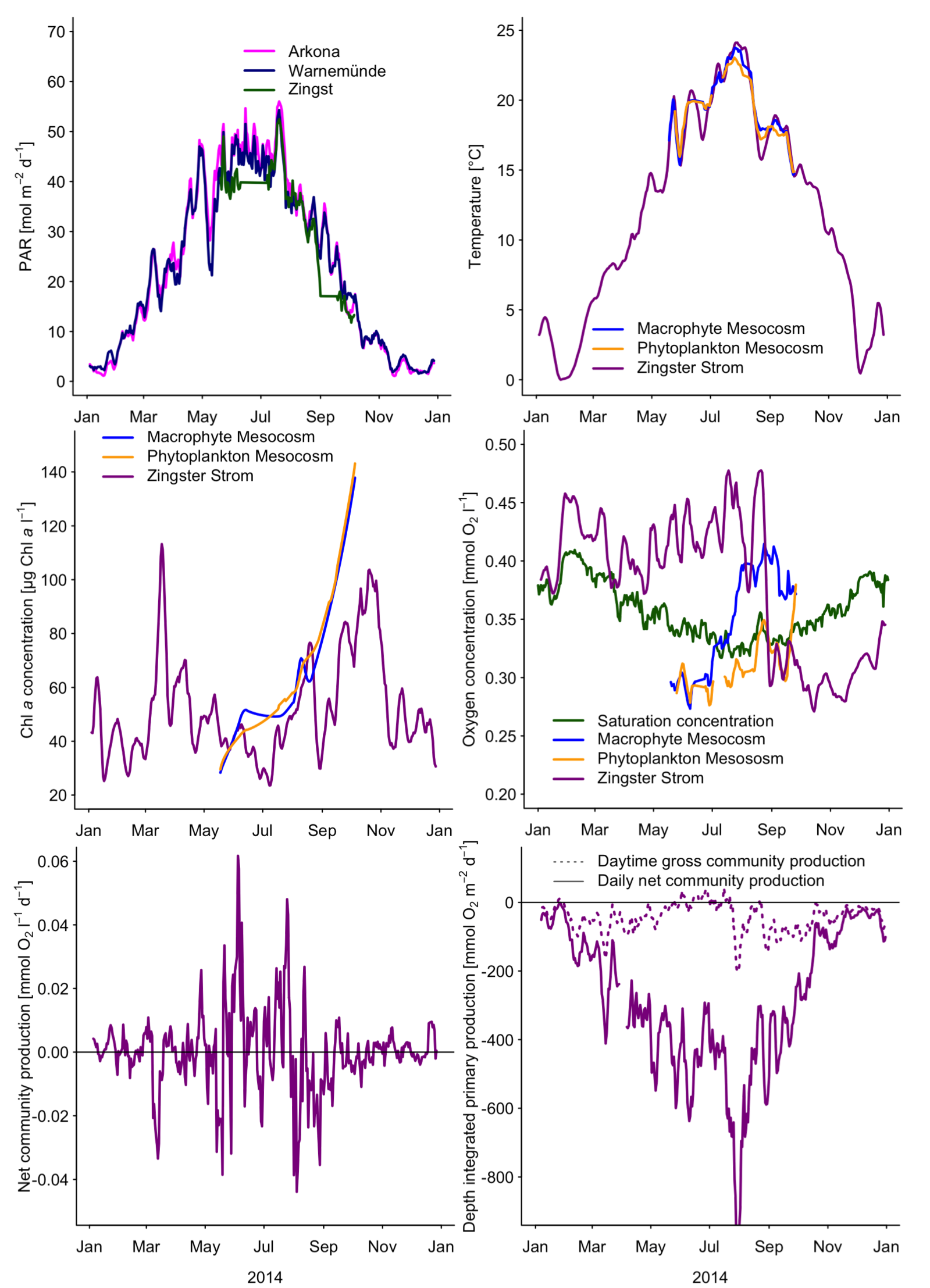

Supplement: S3 Fig — Blue line–macrophyte mesocosm, yellow line–phytoplankton mesocosm, magenta–Zingster Strom. Depth-integrated daily net community production (mmol O2 m-2 d-1), the sum of daytime production and night time respiration, and daytime gross community production (mmol O2 m-2 d-1), calculated as the integrated sum of net daytime production and a daytime respiration demand. Daytime respiration was calculated from the average night time respiration rate of the respective system and multiplied by the hours of daylight. Dates of cleaning and sampling in the mesocosms including the following day were removed prior the analysis. Please note the different scaling. (TIF) [file pone.0247696.s005.tif]
