# Supplementary material for: Dynamics of primary productivity in relation to submerged vegetation of a shallow, eutrophic lagoon: A field and mesocosm study
Source: PLoS One. 2021 May 6;16(5):e0247696. doi: 10.1371/journal.pone.0247696 (PMC8101763; doi:10.1371/journal.pone.0247696)
Supplement: S1 Table — Parameters include dissolved inorganic phosphorus (DIP) and dissolved inorganic nitrogen (DIN, sum of ammonium, nitrate and nitrite) from July to September. Standard deviation (±) was based on n = 3 in mesocosms and n = 30–31 in the Zingster Strom. (DOCX) [file pone.0247696.s006.docx]

S1Table

|  | DIP (µmol l^-1^) | | | DIN (µmol l^-1^) | | |
| --- | --- | --- | --- | --- | --- | --- |
|  | July | August | September | July | August | September |
| Macrophyte mesocosms | 0.01±0.01 | 0±0.01 | 0.01±0.01 | 2.6±0.2 | 1.9±0.4 | 0.7±0.1 |
| Phytoplankton mesocosms | 0.07±0.1 | 0.0±0.0 | 0.05±0.04 | 2.75±1.0 | 1.4±0.5 | 0.13±0.09 |
| Zingster Strom | 0.07±0.2 | 0±0.3 | 0.13±0.3 | 1.4±1.3 | 0.9±1.1 | 0.7±0.7 |
