# Supplementary material for: Dynamics of primary productivity in relation to submerged vegetation of a shallow, eutrophic lagoon: A field and mesocosm study
Source: PLoS One. 2021 May 6;16(5):e0247696. doi: 10.1371/journal.pone.0247696 (PMC8101763; doi:10.1371/journal.pone.0247696)
Supplement: S2 Table — Parameters include r chlorophyll a (μg l-1), particulate organic carbon (POC, mg l-1), particulate organic nitrogen (PON, mg l-1), Carbon: Chlorophyll a (C/Chl a, mol C g Chl a-1), share of POC on total particulate matter (POC: seston in % based on mg l-1) in macrophyte mesocosms, phytoplankton mesocosms and the Zingster Strom. (DOCX) [file pone.0247696.s007.docx]

S2 Table

|  |  | Macrophyte Mesocosm | Phytoplankton mesocosm | Zingster Strom |
| --- | --- | --- | --- | --- |
| Chl *a*  (µg l^-1^) | August | 52.4 – 95.2 | 31 – 128.5 | 24.8 – 92.5 |
|  | October | 37.9 – 277.1 | 56 – 256.6 | 47 – 121 |
| POC  (mg l^-1^) | August | 18.5 – 20.3 | 7.8 – 31.8 | 9.3 – 16.5 |
|  | October | 13.2 – 57.7 | 13.9 – 73.3 | 4.3 – 10.5 |
| PON  (mg l^-1^) | August | 1.6 – 2.2 | 1.2 – 3.2 | 1.1 – 1.8 |
|  | October | 2.6 – 5.4 | 1.8 – 5.7 | 0.7 – 1.3 |
| POC/Chl *a*  (mol g^-1^) | August | 16.4 – 29.5 | 19.8 – 21 | 15.8 – 20.9 |
|  | October | 15.4 – 28.9 | 11.4 – 23.8 | 6.1 – 8.8 |
| POC:Seston  (%) | August | 24.4 – 28.4 | 23.9 – 28.5 | 19 – 35 |
|  | October | 24.4 – 27.2 | 24.5 – 26.8 | 28 – 37 |
